# Curcumin Attenuates Asthmatic Airway Inflammation and Mucus Hypersecretion Involving a PPAR*γ*-Dependent NF-*κ*B Signaling Pathway In Vivo and In Vitro

**DOI:** 10.1155/2019/4927430

**Published:** 2019-04-03

**Authors:** Tao Zhu, Zhihong Chen, Guihua Chen, Daoxin Wang, Shuo Tang, Huojin Deng, Jing Wang, Shengjin Li, Jian Lan, Jin Tong, He Li, Xinyu Deng, Wei Zhang, Jiayang Sun, Yuesheng Tu, Wanting Luo, Changyi Li

**Affiliations:** ^1^Respiratory Medicine, Second Affiliated Hospital of Chongqing Medical University, 400010 Chongqing, China; ^2^Respiratory Medicine, Zhongshan Hospital, Fudan University, Shanghai 20032, China; ^3^Orthopedics Medicine, The Eighth Affiliated Hospital of Sun Yat-sen University, Shenzhen 517000, China; ^4^Respiratory Medicine, Zhujiang Hospital, Southern Medical University, Guangzhou 510280, China; ^5^Respiratory Medicine, First Affiliated Hospital of Chengdu Medical College, Chengdu, 610500 Sichuan, China; ^6^Respiratory Medicine, Affiliated Hospital of Guiyang Medical University, 550004 Guiyang, China; ^7^Southern Medical University, Guangzhou 510280, China

## Abstract

Asthma is characterized by airway inflammation and mucus hypersecretion. Curcumin possessed a potent anti-inflammatory property involved in the PPAR*γ*-dependent NF-*κ*B signaling pathway. Then, the aim of the current study was to explore the value of curcumin in asthmatic airway inflammation and mucus secretion and its underlying mechanism. In vivo, mice were sensitized and challenged by ovalbumin (OVA) to induce chronic asthma. Airway inflammation and mucus secretion were analyzed. In vitro, BEAS-2B cells were obtained. MCP-1, MUC5AC, and PPAR*γ* expression and the phosphorylation of NF-*κ*B p65 and NF-*κ*B p65 DNA-binding activity were measured in both the lungs and BEAS-2B cells. shRNA-PPAR*γ* was used to knock down PPAR*γ* expression. We found that OVA-induced airway inflammation and mucus hypersecretion in mice, OVA and IL-4-induced upregulation of MCP-1 and MUC5AC, suppression of PPAR*γ*, and activation and translocation of NF-*κ*B p65 were notably improved by curcumin both in vivo and in vitro. Our data also showed that these effects of curcumin were significantly abrogated by shRNA-PPAR*γ*. Taken together, our results indicate that curcumin attenuated OVA-induced airway inflammation and mucus hypersecretion in mice and suppressed OVA- and IL-4-induced upregulation of MCP-1 and MUC5AC both in vivo and in vitro, most likely through a PPAR*γ*-dependent NF-*κ*B signaling pathway.

## 1. Introduction

Airway inflammation and mucus hypersecretion are two of the most important characteristics of chronic asthma [1–3]. The recruitment of a variety of inflammatory cells, including lymphocytes, eosinophils, neutrophils, and macrophages, into the airway is considered as a crucial event in the maintenance and development of asthma [1, 2, 4]. Many studies confirmed that a broad spectrum of inflammatory mediators, such as IL-4, IL-5, and IL-13, synthesized and released by activated inflammatory cells, particularly Th2 cells, eventually induce airway mucus hypersecretion which is essential for the pathogenesis of asthma [3, 5]. Several studies showed that extensive mucus plug accumulation inside of the airway lumen was commonly observed in patients with severe asthma and fatal asthma, leading to severe airway obstruction and respiratory failure [6, 7]. Green et al. showed that bronchial mucous glands, resulting in mucous plugs within the airway lumen and ectasia of the gland ducts, are increased in fatal asthma and may contribute to asphyxia due to mucous plugging in the autopsy, compared with nonfatal asthma and nonasthma [7]. However, at present, there is still no effective treatment for airway mucus hypersecretion. Therefore, new therapeutic treatments for this unmet medical need are meaningful and valuable.

Curcumin, a natural polyphenolic compound isolated from the plant turmeric, showed a potent anti-inflammatory activity in many diseases, such as hepatic fibrosis, neurodegenerative diseases, cerebral injury, diabetes, and rheumatoid arthritis [8–12]. Previous studies have shown that the anti-inflammatory properties of curcumin are involved in the regulation of PPAR*γ* [13–16]. Liu et al. found that curcumin reduced PDGFR-*β* expression and inhibited cell proliferation and differentiation in TGF-*β*-induced mouse lung fibroblasts through upregulation of PPAR*γ* [14]. Meng et al. showed that cardiac fibrosis in spontaneously hypertensive rats (SHRs) and angiotensin II- (Ang II-) induced production of CTGF, PAI-1, and ECM in rat cardiac fibroblasts were alleviated by curcumin via a PPAR*γ*-dependent signaling pathway [16]. Simultaneously, some studies found that curcumin could attenuate airway inflammation in asthmatic animal models [17–21]. Subhashini et al. demonstrated that OVA-induced airway inflammation was reduced by curcumin involved in the suppression of p38 MAPK, ERK 42/44, and JNK 54/56 activation in mice [20]. Liu et al. found that curcumin could attenuate airway inflammation possibly through the inactivating Nrf2/HO-1 signaling pathway in mice [19]. However, the underlying mechanism is still in controversy. Additionally, the studies confirmed that PPAR*γ*, a ligand-activated transcription factor, plays a critical role in the mediation of inflammatory mediator release and inflammatory cell activation in asthma in both human and animals [22–25]. Xu et al. reported that OVA-induced airway inflammation and airway remodeling were improved by PPAR*γ* agonist rosiglitazone in mice [25]. Huang et al. showed that TNF-*α*-induced ICAM1 gene expression was inhibited by PPAR*γ* agonist ciglitazone in human airway smooth muscle (ASM) cells [24]. Therefore, the purpose of our current study was to explore whether curcumin could alleviate OVA and IL-4-induced asthmatic airway inflammation and mucus hypersecretion both in vivo and in vitro and its involved signal pathway.

## 2. Material and Methods

### 2.1. Animals

All procedures involving animals were approved by the Animal Experimental Ethics Committee of Chongqing Medical University (No. 2018-199). The current study was performed according to the recommendations of the Guide for the Care and Use of Laboratory Animals. All surgery was performed using sodium pentobarbital anesthesia, and all efforts were made to minimize suffering. Specific pathogen-free male BALB/c mice (18-22 g, age about 6-8 weeks) were maintained under specific pathogen-free conditions in the animal center facilities of our university. The mice were kept in a temperature-controlled room (12-hour dark and light cycles) and offered *ad libitum* access to food and water. Animals underwent an acclimatization period of at least 1 week before the study.

### 2.2. Knockdown of PPAR*γ* by shRNA in Mice

To silence the expression of PPAR*γ* in the lung, the recombinant lentivirus vector for PPAR*γ* (shRNA-PPAR*γ*, sc-29456-V, Santa Cruz Biotechnology, CA, USA) was used. And a negative control lentivirus-expressing nontargeting sequence (sc-108080, Santa Cruz Biotechnology, CA, USA) was obtained as shRNA-scrambled. Briefly, thirty male BALB/c mice were divided into 3 groups, control group, shRNA-scrambled group, and shRNA-PPAR*γ* group, with 10 mice in each [26]. After anesthesia, the shRNA-PPAR*γ* lentivirus vector (40 *μ*L) for the shRNA-PPAR*γ* group or negative control lentivirus (40 *μ*L) for the shRNA-scrambled group was given by intratracheal injection. Then, the mice in the control group were administrated sterile saline instead. Twenty days after transfection, the left lower lung was resected. Histology changes were observed by H&E staining. The efficiency of shRNA transfection was measured by qPCR and western blotting analysis.

### 2.3. A Murine Model of OVA-Induced Chronic Asthma

Fifty male BALB/c mice were randomly divided into 5 groups: control group, curcumin (Cur) group, OVA group, OVA + Cur group, and OVA + Cur + shRNA-PPAR*γ* group, with 10 mice in each. According to our previous studies, a murine model of chronic asthma was induced by OVA sensitization and challenge [3, 4]. Briefly, mice were sensitized intraperitoneally with 10 *μ*g of OVA (grade V, Sigma-Aldrich Chemical, St. Louis, MO, USA) in 100 *μ*g of Al (OH)_3_ on days 0, 7, and 14. From days 15 to 75, the mice were challenged with 5% OVA aerosol for 1.5 h once daily. Meanwhile, from days 15 to 75, curcumin (100 mg/kg, 08511, Sigma-Aldrich, USA) was given by gastric lavage daily [27, 28]. shRNA-PPAR*γ* (40 *μ*L) was also given by intratracheal injection on days 15, 35, and 55.

### 2.4. Cell Counts in BALF

One hour after OVA challenge on day 75, mice were sacrificed after anesthesia by pentobarbital (50 mg/kg i.p.). According to our previous studies, BALF was collected [3, 4, 29]. And the fluid recovery rate was about 85%-90%. Then, total cells, neutrophils, macrophages, eosinophils, and lymphocytes were counted double-blindly with a hemocytometer [4].

### 2.5. TNF-*α*, IL-4, IL-5, and IL-13 in BALF

According to our previous studies, the BALF supernatant was collected after centrifugation and stored at –80°C before cytokine assay [3, 4, 29]. TNF-*α*, IL-4, IL-5, and IL-13 in BALF were measured by ELISA (R&D Systems, USA).

### 2.6. H&E Staining and Periodic Acid-Schiff (PAS) Staining

The right lower lung of each mouse was fixed in 10% formalin, embedded in paraffin, cut into 5 *μ*m sections, and stained with H&E to observe the pathological alterations of the lung. Meanwhile, PAS staining was performed to observe goblet cell hyperplasia and airway mucus secretion. According to our previous studies, the histological mucus index (the percentage of the mucus-positive area of the whole bronchial epithelium) was recorded [3, 4, 29].

### 2.7. Cell Transfection with shRNA

BEAS-2B cells, a cell line derived from human bronchial epithelial cells, were cultured in DMEM supplemented with penicillin (100 IU/mL), streptomycin (100 *μ*g/mL), and 10% heat-inactivated fetal bovine serum (FBS). BEAS-2B cells were cultured in an incubator maintained with 5% CO_2_ at 37°C. Then, BEAS-2B cells were transfected at 70% confluence with shRNA-PPAR*γ* (sc-44220-V, Santa Cruz Biotechnology, CA, USA) or shRNA-scrambled (sc-108060, Santa Cruz Biotechnology, CA, USA). Twenty-four hours after transfection, BEAS-2B was used for further experiments [30]. Meanwhile, knockdown of PPAR*γ* expression was analyzed by qPCR and western blotting.

### 2.8. Cell Treatment

shRNA-PPAR*γ* transfected and nontransfected BEAS-2B cells were pretreated with 5 *μ*M curcumin (0.1% DMSO) [31], 4 h before IL-4 (20 ng/mL, R&D Systems, Minneapolis, USA) stimulation [32]. Twenty-four hours after interventions, MTT assay was performed to assess cell viability [30, 33]. Meanwhile, total and nuclear proteins and mRNA were extracted from cells and kept at -80°C.

### 2.9. Quantitative PCR

The mRNA expressions of MCP-1, MUC5AC, and PPAR*γ* were measured by qPCR [4, 30]. *β*-Actin was used as an internal reference. Briefly, the right upper lung tissues were kept at -80°C. Total RNA was isolated from the lung, and BEAS-2B cells by TRIzol reagent (Invitrogen, USA). The PrimeScript® RT reagent kit with gDNA eraser (Takara Bio Inc., Otsu, Japan) was used for reverse transcription. PCR was then performed with the iQ™5 Multicolor Real-Time PCR Detection System (Bio-Rad Laboratories, Inc., USA) and an SYBR Green PCR kit (Takara Bio Inc., Otsu, Japan) in a final volume of 20 *μ*L, containing 1.6 *μ*L cDNA template, forward and backward primers (0.8 *μ*L each), 10 *μ*L SYBR® Premix Ex Taq™ II, and 6.8 *μ*L dH_2_O. The primers and TaqMan probes were designed using Primer Premier (PREMIER Biosoft International, Canada). The premier sequences were as follows: mMCP-1 (forward) 5′-TTAAAAA CCTGG ATCGGA ACCAA-3′ and (reverse) 5′-GCATTAGC TTCAG ATTTAC GGGT-3′; mMUC5AC (forward) 5′-GATGACTTCCAGACTATCAGTG-3′ and (reverse) 5′-TGGCGTTAGTCAGCAGA-3′; mPPAR*γ* (forward) 5′-TTTTCA AGGGT GCCAG TTTC-3′ and (reverse) 5′-TTATTC ATCAGG GAGG CCAG-3′; m*β*-actin (forward) 5′-GATTACTGCTCTGGCTCCTAGC-3′ and (reverse) 5′-ACTCATCGTAC TCCTGCTTGCT-3′; hMCP-1: (forward) 5′-CCC CAGTCACCTGCT GTTAT-3′ and (reverse) 5′-CCACA ATGGTCTTGAA GATCA C-3′; hMUC5AC: (forward) 5′-ATTTTT TCCCCACTCC TGATG-3′ and (reverse) 5′-AAGACA ACCCAC TCCCAACC-3′; hPPAR*γ* (forward) 5′-GCCAT TTTCTC AAACGAG AGTCAGC-3′ and (reverse) 5′-CCACGG AGCTGATC CCAA AGTT-3′; and h*β*-actin: (forward) 5′-CTTAGTT GCGTTA CACC CTTTCTTG-3′ and (reverse) 5′-CTGTCAC CTTCA CCGTTC CAGTTT-3′. Changes in the expression of target genes were calculated using the 2^-ΔΔCt^ method, ΔΔCt = (Ct_target_ − Ct_*β*‐actin_)_sample_ − (Ct_target_ − Ct_*β*‐actin_)_control_.

### 2.10. Western Blotting

Western blotting was performed to evaluate the protein expression [34, 35]. Briefly, protein lysates from the left upper lung tissues and BEAS-2B cells were subjected to sodium dodecyl sulfate-polyacrylamide gel electrophoresis and then transferred to nitrocellulose membranes. Antibodies against mMCP-1, mMUC5AC, mPPAR*γ*, mphospho-NF-*κ*B p65, mNF-*κ*B p65, m*β*-actin, hMCP-1, hMUC5AC, hPPAR*γ*, hphospho-NF-*κ*B p65, hNF-*κ*B p65, and h*β*-actin were purchased from Santa Cruz Biotechnology (Santa Cruz, CA, USA). The relative protein levels of mMCP-1, mMUC5AC, mPPAR*γ*, hMCP-1, hMUC5AC, and hPPAR*γ* were normalized to that of *β*-actin. The levels of mphospho-NF-*κ*B p65 and hphospho-NF-*κ*B p65 in each sample were measured as ratios of intensities of phospho-NF-*κ*B p65 to total NF-*κ*B p65 bands.

### 2.11. NF-*κ*B p65 DNA-Binding Activity Assay

NF-*κ*B p65 DNA-binding activity was measured by TransAM™ NF-*κ*B p65 Chemi Transcription Factor Assay kit (Active Motif, Carlsbad, CA, USA) in our study [4, 30, 33].

### 2.12. Statistical Analysis

Statistical analyses were performed with SPSS software, version 17.0 (SPSS Inc., Chicago, IL, USA). All data were presented as mean ± standard error of the mean (SEM). One-way analysis of variance (ANOVA) was performed. *P* < 0.05 was considered to be statistically significant.

## 3. Results

### 3.1. PPAR*γ* Expression Was Suppressed after Transfection of the Lung with shRNA-PPAR*γ*

**Figure 1:**
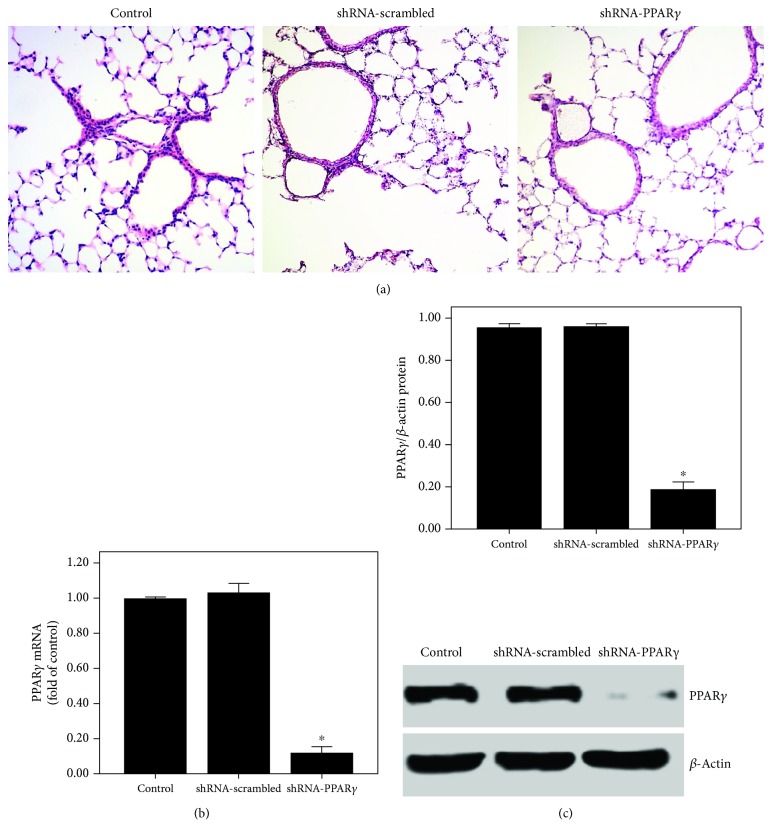
PPAR*γ* expression was inhibited after transfection of the lung with shRNA-PPAR*γ*. Mice were transfected with shRNA-PPAR*γ* or shRNA-scrambled by intratracheal injection. Twenty days after transfection, the expression of PPAR*γ* was measured. (a) After 20 days of transfection, mice were sacrificed and their left lower lungs were fixed. The tissue sections were then stained with H&E. The figure demonstrates a representative view (×200) from each group. (b) qPCR was used to analyze the mRNA expression of PPAR*γ*. (c) Western blotting was performed to evaluate the protein expression of PPAR*γ*. Each bar represents the mean ± SEM of 10 mice. ^∗^*P* < 0.05 compared with control.

After 20 days of transfection, no pathological changes were observed in the three groups of mice (Figure 1(a)). However, PPAR*γ* expression was significantly suppressed by shRNA-PPAR*γ* in the lung tissues (Figures 1(b) and 1(c)).

### 3.2. Curcumin Attenuated Pulmonary Pathological Alterations in OVA-Induced Chronic Asthma

**Figure 2:**
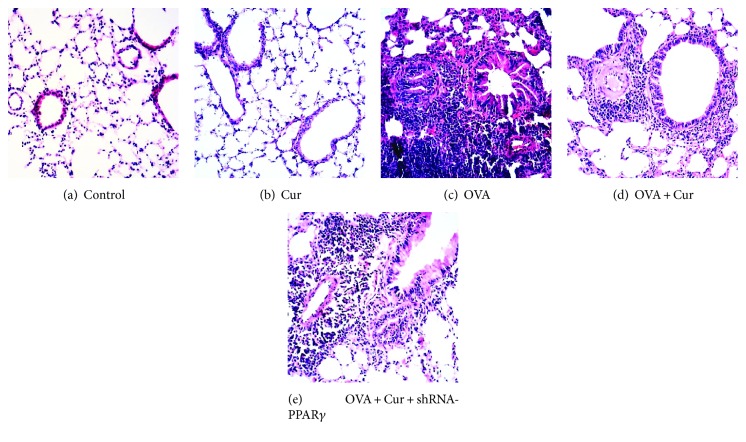
Curcumin attenuated pulmonary pathological alterations in OVA-induced chronic asthma. After 75 days of OVA sensitization and challenge, mice were sacrificed and their right lower lungs were fixed. Then, tissue sections were stained with hematoxylin and eosin (H&E). The figure demonstrates a representative view (×200) from each group.

After 75 days of OVA sensitization and challenge, the severe and classical pathological alterations were found, including extensive inflammatory cell infiltration in the airway, goblet cell hyperplasia, smooth muscle hyperplasia and hypertrophy, collagen deposition, and thickening of the airway basement membrane (Figure 2). However, OVA-induced airway inflammation was markedly inhibited by curcumin (Figure 2). Furthermore, this effect of curcumin was significantly blunted by shRNA-PPAR*γ*. No pathological alterations were observed in the control and curcumin groups (Figure 2).

### 3.3. Curcumin Inhibited Inflammatory Cell Counts and Inflammatory Mediators in BALF in OVA-Induced Chronic Asthma

**Figure 3:**
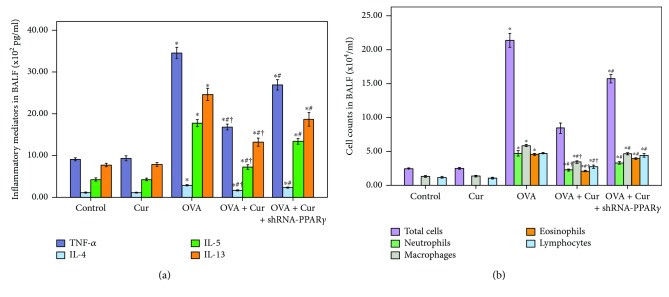
Curcumin inhibited inflammatory cell counts and inflammatory mediators in BALF in OVA-induced chronic asthma. (a) TNF-*α*, IL-4, IL-5, and IL-13 in BALF were detected by ELISA. (b) Cells in BALF were collected, and cytospin preparations were made. Total cells, neutrophils, macrophages, eosinophils, and lymphocytes in BALF were measured; each bar represents the mean ± SEM of 10 mice. ^∗^*P* < 0.05 compared with control. ^#^*P* < 0.05 compared with OVA. ^†^*P* < 0.05 compared with OVA + Cur + shRNA-PPAR*γ*.

In our study, cell counts and inflammatory mediators, including TNF-*α*, IL-4, IL-5, and IL-13, in BALF was measured to evaluate the severity of airway inflammation in the lungs. As shown in Figure 3, OVA induced the increase in total cells, neutrophils, macrophages, eosinophils, and lymphocytes, and increased levels of TNF-*α*, IL-4, IL-5, and IL-13 in BALF were remarkably reduced by curcumin. Meanwhile, our data also demonstrated that these effects of curcumin were markedly abrogated by shRNA-PPAR*γ* (Figure 3).

### 3.4. Curcumin Reduced the MCP-1 Expression in the Lung in OVA-Induced Chronic Asthma

**Figure 4:**
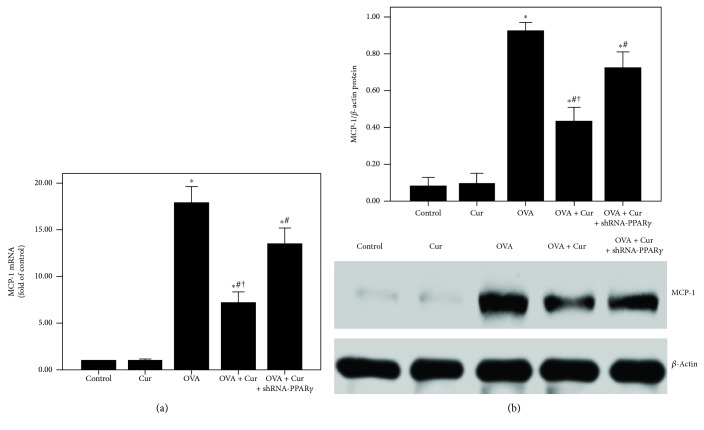
Curcumin reduced the MCP-1 expression in the lung in OVA-induced chronic asthma. (a) qPCR was used to measure MCP-1 mRNA expression in the lung; (b) western blotting was performed to measure MCP-1 protein expression in the lung. Each bar represents the mean ± SEM of 10 mice. ^∗^*P* < 0.05 compared with control. ^#^*P* < 0.05 compared with OVA. ^†^*P* < 0.05 compared with OVA + Cur + shRNA-PPAR*γ*.

To explore the mRNA and protein expression of MCP-1, qPCR and western blotting were obtained in the current study. As shown in Figure 4, OVA-induced upregulation of MCP-1 was noticeably decreased by curcumin. Furthermore, this effect of curcumin was largely blocked by shRNA-PPAR*γ*.

### 3.5. Curcumin Suppressed Airway Mucus Secretion in OVA-Induced Chronic Asthma

**Figure 5:**
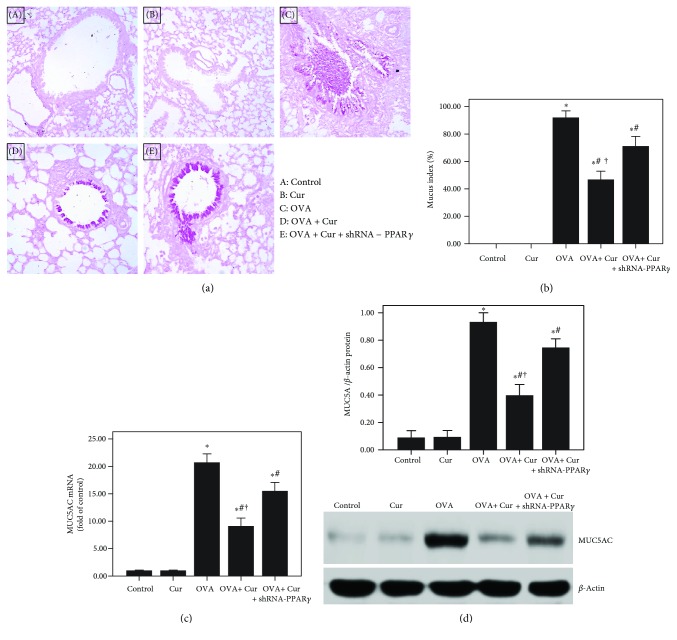
Curcumin suppressed airway mucus secretion in OVA-induced chronic asthma. (a) After 75 days of OVA sensitization and challenge, mice were sacrificed and their right lower lungs were fixed. Then, tissue sections were stained with periodic acid-Schiff (PAS) staining. The figure demonstrates a representative view (×200) from each group; (b) mucus index was calculated as the percentage of the mucus-positive area of the whole bronchial epithelium; (c) qPCR was performed to measure MUC5AC mRNA expression in the lung; (d) western blotting was obtained to analyze MUC5AC protein expression in the lung. Each bar represents the mean ± SEM of 10 mice. ^∗^*P* < 0.05 compared with control. ^#^*P* < 0.05 compared with OVA. ^†^*P* < 0.05 compared with OVA + Cur + shRNA-PPAR*γ*.

As shown in Figures 5(a) and 5(b), OVA-induced mucus hypersecretion and goblet cell hyperplasia were remarkably alleviated by curcumin. And our result also found that OVA-induced upregulation of MUC5AC was markedly reduced by curcumin (Figures 5(c) and 5(b)). Furthermore, these effects of curcumin were significantly blunted by shRNA-PPAR*γ*.

### 3.6. Curcumin Inhibited OVA-Induced NF-*κ*B Activation and DNA-Binding Activity through Upregulation of PPAR*γ* in the Lung in OVA-Induced Chronic Asthma

**Figure 6:**
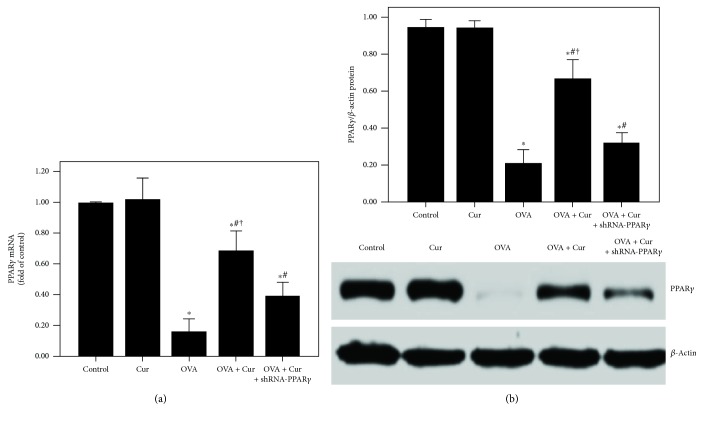
Curcumin upregulated the PPAR*γ* expression in the lung in OVA-induced chronic asthma. (a) qPCR was used to measure PPAR*γ* mRNA expression in the lung; (b) western blotting was performed to measure PPAR*γ* protein expression in the lung. Each bar represents the mean ± SEM of 10 mice. ^∗^*P* < 0.05 compared with control. ^#^*P* < 0.05 compared with OVA. ^†^*P* < 0.05 compared with OVA + Cur + shRNA-PPAR*γ*.

**Figure 7:**
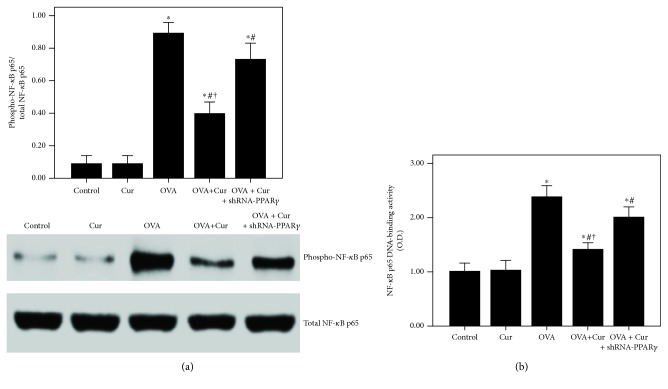
Curcumin inhibited OVA-induced NF-*κ*B activation and DNA-binding activity in the lung. (a) Western blotting was performed to analyze the phosphorylation of NF-*κ*B p65 in the lung; (b) DNA-binding activity of NF-*κ*B p65 was measured by a TransAM™ p65 transcription factor ELISA kit. Each bar represents the mean ± SEM of 10 mice. ^∗^*P* < 0.05 compared with control. ^#^*P* < 0.05 compared with OVA. ^†^*P* < 0.05 compared with OVA + Cur + shRNA-PPAR*γ*.

Our data showed that OVA-induced downregulation of PPAR*γ* was notably abrogated by curcumin in the lung (Figure 6). Subsequently, as shown in Figure 7, OVA-induced activation of NF-*κ*B p65 and increase in NF-*κ*B p65 DNA-binding activity were remarkably inhibited by curcumin in the lung. Furthermore, these effects of curcumin were markedly blunted by shRNA-PPAR*γ*.

### 3.7. Curcumin Was Nontoxic to BEAS-2B Cells

**Figure 8:**
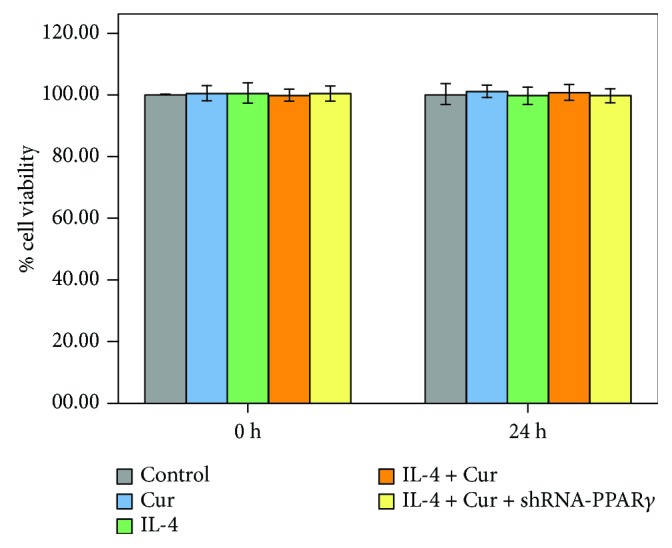
Curcumin was nontoxic to BEAS-2B cells. shRNA-transfected or nontransfected BEAS-2B cells were treated with DMSO, curcumin (5 *μ*M), and IL-4 for 24 h. MTT assay was performed to assess the cell viability of BEAS-2B cells. Quantitative data were presented as mean ± SEM (*n* = 5). ^∗^*P* < 0.05 compared with control.

As shown in Figure 8, no significant difference in cell viability was found in different groups after 24 h of interventions.

### 3.8. PPAR*γ* Expression Was Suppressed after shRNA-PPAR*γ* Transfection in BEAS-2B Cells

**Figure 9:**
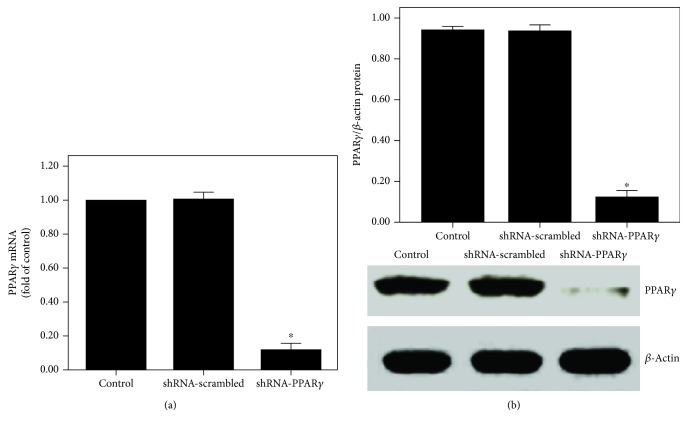
PPAR*γ* expression was inhibited after shRNA-PPAR*γ* transfection in BEAS-2B cells. BEAS-2B cells were transfected with shRNA-PPAR*γ* or shRNA-scrambled. Twenty-four hours after transfection, PPAR*γ* expression was analyzed. (a) qPCR was performed to measure PPAR*γ* mRNA expression. (b) Western blotting was obtained to evaluate PPAR*γ* protein expression. Quantitative data were presented as mean ± SEM (*n* = 5). ^∗^*P* < 0.05 compared with control. ^#^*P* < 0.05 compared with IL-4. ^†^*P* < 0.05 compared with IL-4 + Cur + shRNA-PPAR*γ*.

After 24 h of transfection, PPAR*γ* expression was noticeably suppressed by shRNA-PPAR*γ* in BEAS-2B cells (Figure 9).

### 3.9. Curcumin Reduced MCP-1 and MUC5AC Expression in IL-4-Induced BEAS-2B Cells

**Figure 10:**
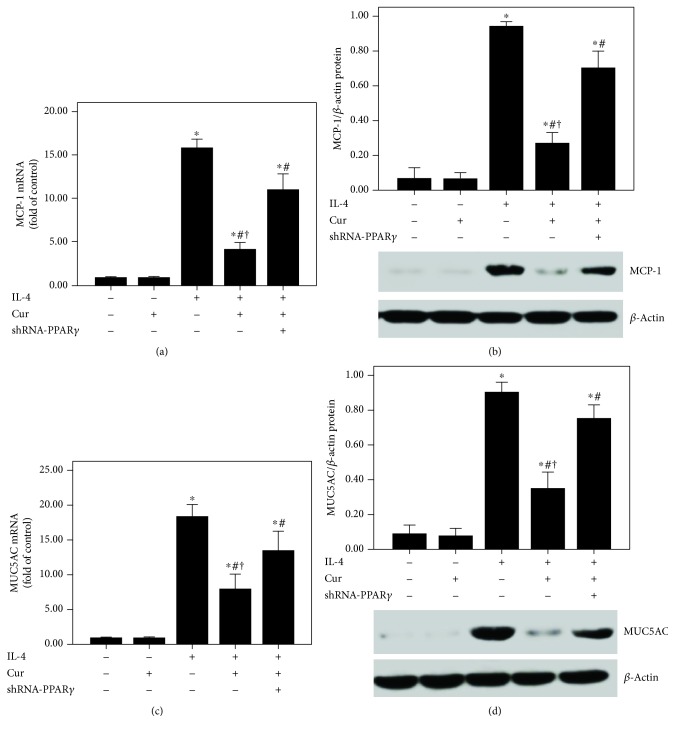
Curcumin suppressed MCP-1 and MUC5AC expression in IL-4-induced BEAS-2B cells. (a and c) qPCR was used to measure MCP-1 and MUC5AC mRNA expression in BEAS-2B cells; (b and d) western blotting was performed to measure MCP-1 and MUC5AC protein expression in BEAS-2B cells. Quantitative data were presented as mean ± SEM (*n* = 5). ^∗^*P* < 0.05 compared with control. ^#^*P* < 0.05 compared with IL-4. ^†^*P* < 0.05 compared with IL-4 + Cur + shRNA-PPAR*γ*.

As shown in Figure 10, IL-4-induced upregulation of MCP-1 and MUC5AC was largely reduced by curcumin. Furthermore, these effects of curcumin were notably abrogated by shRNA-PPAR*γ*.

### 3.10. Curcumin Inhibited IL-4-Induced NF-*κ*B Activation and DNA-Binding Activity through Upregulation of PPAR*γ* in BEAS-2B Cells

**Figure 11:**
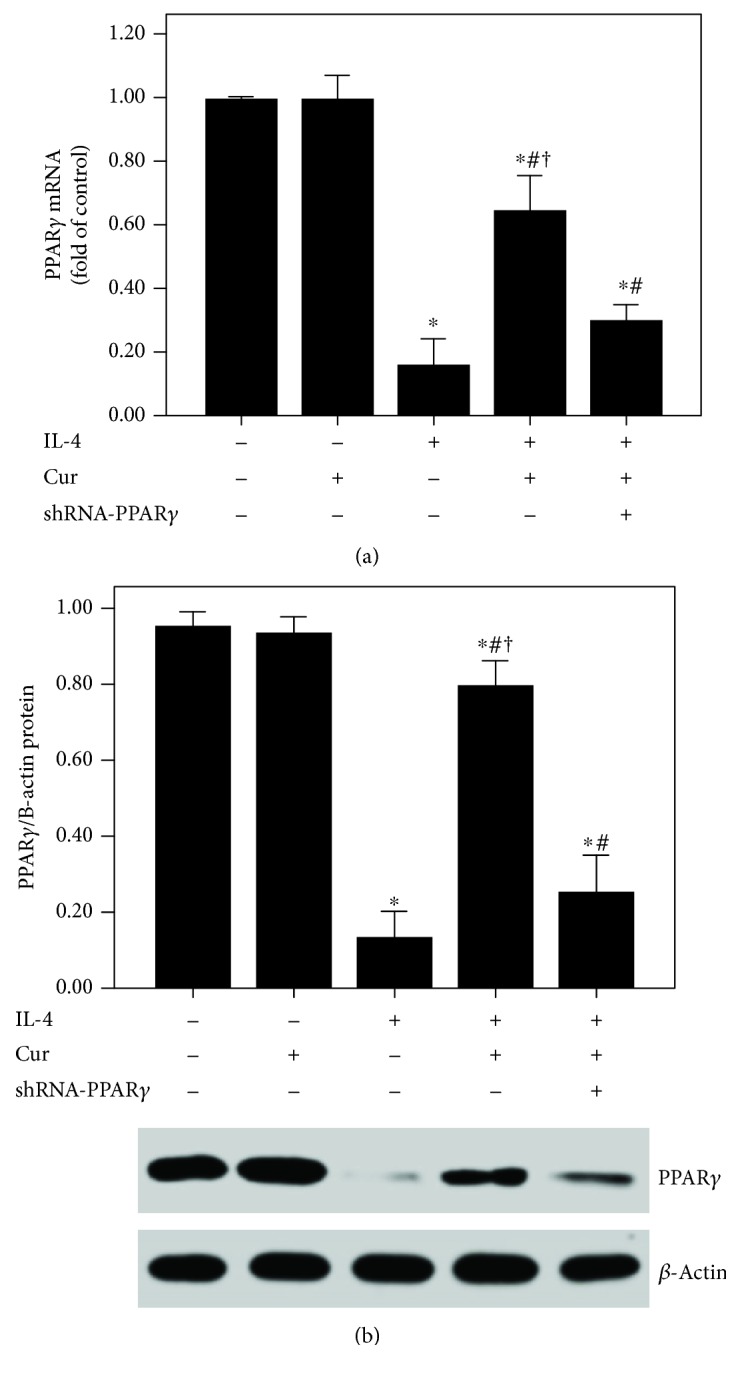
Curcumin upregulated the PPAR*γ* expression in IL-4-induced BEAS-2B cells. (a) qPCR was used to measure PPAR*γ* mRNA expression in BEAS-2B cells; (b) western blotting was performed to measure PPAR*γ* protein expression in BEAS-2B cells. Quantitative data were presented as mean ± SEM (*n* = 5). ^∗^*P* < 0.05 compared with control. ^#^*P* < 0.05 compared with IL-4. ^†^*P* < 0.05 compared with IL-4 + Cur + shRNA-PPAR*γ*.

**Figure 12:**
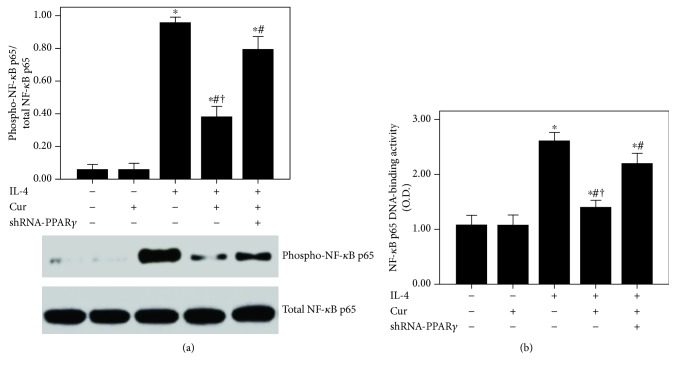
Curcumin inhibited IL-4-induced NF-*κ*B activation and DNA-binding activity in BEAS-2B cells. (a) Western blotting was performed to analyze the phosphorylation of NF-*κ*B p65 in BEAS-2B cells; (b) DNA-binding activity of NF-*κ*B p65 was measured by a TransAM™ p65 transcription factor ELISA kit. Quantitative data were presented as mean ± SEM (*n* = 5). ^∗^*P* < 0.05 compared with control. ^#^*P* < 0.05 compared with IL-4. ^†^*P* < 0.05 compared with IL-4 + Cur + shRNA-PPAR*γ*.

According to our data, the expression of PPAR*γ* was significantly inhibited after 24 h of IL-4 administration (Figure 11). Curcumin markedly enhanced the PPAR*γ* expression in BEAS-2B cells after IL-4 administration (Figure 11). Subsequently, IL-4-induced activation of NF-*κ*B p65 and increase in NF-*κ*B p65 DNA-binding activity were also substantially abrogated by curcumin in BEAS-2B cells (Figure 12). Furthermore, these effects of curcumin were largely blocked by shRNA-PPAR*γ*.

## 4. Discussion

In the current study, our data suggested that OVA-induced airway inflammation and airway mucus hypersecretion in mice were remarkably alleviated and OVA- and IL-4-induced upregulation of MCP-1 and MUC5AC in both lung and BEAS-2B cells was notably suppressed by curcumin. Furthermore, our data also indicated that the anti-inflammatory and airway mucus secretion inhibitory property of curcumin was most likely mediated through a PPAR*γ*-dependent NF-*κ*B signaling pathway.

Asthma is a very common chronic airway disorder, featured with reversible airflow obstruction, all over the world. It is estimated that the prevalence of asthma is about 1% to 18% in different counties and regions [36, 37]. It is well-known that airway inflammation plays a hub role in the pathogenesis of asthma [4]. Therefore, anti-inflammatory therapy has been fundamental in asthma treatment.

Turmeric, a widely used herb in many Asian countries, has long been commonly used in a variety of diseases, including rheumatoid arthritis, diarrhea, upper airway infection, and hepatitis. Curcumin, the main component of the yellow color of turmeric, is a natural polyphenol with potent anti-inflammatory and antioxidative effects in different conditions [38, 39]. Sorrenti et al. found that LPS-induced acute brain inflammation and long-term memory impairment were noticeably attenuated by curcumin in mice [38]. Wang et al. showed that ventilator-induced lung injury and inflammation were alleviated by curcumin through inactivation of NF-*κ*B in rats [39]. In the current study, the severe and classical pathological alterations in the lungs were found, after 75 days of OVA sensitization and challenge (Figure 2). Our data demonstrated that OVA-induced pathological alterations in the lungs were compromised by curcumin. It is well-known that inflammatory cells, including macrophages, neutrophils, eosinophils, and lymphocytes, play critical roles in the pathogenesis of asthma [1, 2]. Our findings showed that OVA-induced increase in macrophages, neutrophils, eosinophils, and lymphocytes in BALF was significantly reduced by curcumin. In addition, a number of studies confirmed that Th2 cytokines, including IL-4, IL-5, and IL-13, and other inflammatory mediators, particularly TNF-*α*, were essential for the recruitment of eosinophils, mast cells, and other inflammatory cells into the airway and promotion of goblet cell metaplasia and hyperplasia, resulting in airway mucus hypersecretion in asthma in both human and animals [1, 2]. Anti-IL-4, anti-IL-5, anti-IL-5R, and anti-IL-13 therapies have been developed and in clinical trials in several countries [40, 41]. In our study, we found that OVA-induced increase in TNF-*α*, IL-4, IL-5, and IL-13 levels in BALF was notably suppressed by curcumin. Therefore, these results indicated that OVA-induced airway inflammation was markedly inhibited by curcumin in mice.

Mucus hypersecretion in the airway is another important feature of chronic asthma, leading to extensive mucus plugs in small airways and bronchodilator resistance [4]. Goblet cell metaplasia and hyperplasia in airway-induced mucin overproduction, in response to many endogenous signaling factors, such as epidermal growth factor (EGF), leukotrienes (LTs), and Th2 cytokines, are widely observed in patients with chronic asthma and severe asthma [3, 4, 42]. According to the previous reports, goblet cells in small airways in patients with severe and refractory asthma remarkably increased (more than 20-fold) compared to healthy subjects [2–4, 42]. In the current study, our data showed that OVA-induced mucus hypersecretion and goblet cell hyperplasia were remarkably alleviated by curcumin (Figures 5(a) and 5(b)). Then, several studies confirmed that MUC5AC is the major component of mucus in the airway in asthma in both human and animals [3, 4, 42]. Therefore, the expression of MUC5AC in the lungs was evaluated in our study. We revealed that OVA-induced upregulation of MUC5AC was markedly reduced by curcumin in mice (Figures 5(c) and 5(d)). Furthermore, we also found that IL-4-induced overexpression of MUC5AC was largely decreased by curcumin in BEAS-2B cells (Figures 10(c) and 10(d)). Then, mounting evidence, consistent with our previous study, confirmed that NF-*κ*B, a widely expressed nuclear transcription factor and one of the most important regulators of inflammation, plays a hub role in the modulation of airway mucus secretion in asthma [4, 29, 43]. Lim et al. proved that 3,19-diacetyl-14-deoxy-11,12-didehydroandrographolide (SRS27) reduced OVA-induced overexpression of MUC5AC in the lungs by inhibition of the NF-*κ*B signaling pathway in a murine model of asthma [43]. And our previous study also showed that OVA-induced airway inflammation and mucus hypersecretion were largely attenuated by glucagon-like peptide-1 (GLP-1) analog, liraglutide, through a protein kinase A- (PKA-) dependent NF-*κ*B signaling pathway in mice [4]. Furthermore, some investigations found that the anti-inflammatory and organ-protective properties of curcumin are associated with inactivation of NF-*κ*B in different inflammatory disorders, such as sepsis, diabetes, and traumatic spinal cord injury [44–46]. Zhong et al. showed that LPS-induced inflammation and oxidative stress in sepsis and liver failure were suppressed by curcumin through inactivation of NF-*κ*B in mice [44]. Jiménez-Flores et al. demonstrated that diabetes-associated inflammation and metabolic disorder in the liver were noticeably attenuated by curcumin via the NF-*κ*B signaling pathway in db/db mice [45]. Zeng et al. figured out that high-fat diet-induced oxidative stress, inflammation, and injury in the heart were significantly compromised by curcumin through inactivation of NF-*κ*B both in vivo and in vitro [46]. Additionally, MCP-1, a CC chemokine also named as chemokine (C–C motif) ligand 2 (CCL2), is an important inflammatory molecule in the airway epithelium in asthma [47]. It has been confirmed that the NF-*κ*B signaling pathway is essential for the regulation of MCP-1 expression in the airway epithelium in inflammatory conditions, including asthma, in both mice and BEAS-2B cells [47–50]. Hwang et al. revealed that l-theanine markedly inhibited OVA-induced airway inflammation and upregulation of MCP-1 in the lungs by inactivation of NF-*κ*B in a murine model of asthma [47]. Huang et al. found that TNF-*α*-induced overexpression of MCP-1 was remarkably reduced by conjugated linoleic acids (CLAs) through blocking NF-*κ*B transcription regulation in BEAS-2B cells [48]. In the current study, firstly, our findings demonstrated that OVA- and IL-4-induced overexpression of MCP-1 was significantly reduced by curcumin in both lungs and BEAS-2B cells (Figures 4, 10(a), and 10(b)). Furthermore, we found that OVA- and IL-4-induced NF-*κ*B p65 phosphorylation and enhanced NF-*κ*B p65 DNA-binding activity were also largely abrogated by curcumin both in vivo and in vitro (Figures 7 and 12). These findings indicated that OVA- and IL-4-induced airway inflammation, airway mucus hypersecretion, and overexpression of MCP-1 were compromised by curcumin possibly through inactivation of NF-*κ*B.

However, the signaling pathway of curcumin in the modulation of NF-*κ*B is still not very clear in asthma. Meanwhile, some investigations found that the NF-*κ*B inactive value of curcumin was possibly in a PPAR*γ*-dependent pathway in many pathological conditions, including pulmonary fibrosis, myocardial infarction, diabetes, and cardiac fibrosis [14, 16, 51]. Meng et al. found that cardiac fibrosis was notably blocked by curcumin through upregulation of PPAR*γ* in spontaneously hypertensive rats (SHRs) [16]. Liu et al. showed that TGF-*β*2-driven differentiation of lung fibroblasts to myofibroblasts was largely suppressed by curcumin through the PPAR*γ* signaling pathway in vitro [14]. Li et al. demonstrated that angiotensin II- (AngII-) induced inflammation and oxidative stress were remarkably suppressed by curcumin via increase in PPAR*γ* expression in vascular smooth muscle cells (VSMCs) [51]. Meanwhile, several studies also figured out that airway inflammation was inhibited by curcumin in asthmatic animal models [19, 20]. However, the underlying mechanism is still unclear. Then, we hypothesized that these effects of curcumin in asthma would result from modulation of PPAR*γ*. According to our data, we found that OVA- and IL-4-induced downregulation of PPAR*γ* was significantly increased by curcumin both in vivo and in vitro (Figures 6 and 11). Furthermore, our data also revealed that the effects of curcumin on OVA-induced airway inflammation and mucus hypersecretion in mice and IL-4-induced overexpression of MCP-1 and MUC5AC in BEAS-2B cells were largely blunted by shRNA-PPAR*γ*. These results indicated that the effects of curcumin were induced by upregulation of PPAR*γ* expression. Nevertheless, the potential mechanism of how curcumin regulates NF-*κ*B activation through PPAR*γ* in the airway epithelium in asthma is still not very clear. Some studies found that PPAR*γ* inhibits activation of NF-*κ*B mainly through two major molecular mechanisms. PPAR*γ* can either combine with the NF-*κ*B p50/NF-*κ*B p65 dimer or activate I*κ*B kinase to repress the degradation of I*κ*B*α* to abolish the activation of NF-*κ*B and its DNA-binding activity in inflammatory processes [30, 52]. Therefore, further study should be carried out to elucidate it.

Many studies found that PPAR*γ* agonists, such as ciglitazone, rosiglitazone, and pioglitazone, exert their anti-inflammatory effects primarily by inhibiting proinflammatory mediators and antagonizing the proinflammatory functions of different cell types relevant to asthma pathophysiology in both human and animals [23–25, 53]. Specifically, PPAR*γ* activation or PPAR*γ* agonists have displayed beneficial effects on multiple asthma features, suppressing airway hyperresponsiveness, reducing inflammatory cell infiltration and epithelial hyperplasia in the airway, decreasing inflammatory mediator synthesis and release, inhibiting collagen deposition, and reducing mucus hypersecretion [23–25, 53, 54]. Some studies also support the potential benefits of PPAR*γ* agonists in the treatment of asthma [55]. Rinne et al. showed that a large number of diabetic patients with asthma found an association between thiazolidinedione (TZD) treatment and decreasing the risk of asthma exacerbations and steroid use in a cohort study [55]. Otherwise, Oh et al. demonstrated that the single-nucleotide polymorphisms (SNPs) in the PPAR*γ* gene (PPARG), +82466C>T, and haplotypes 1(CC) and 2(CT) were associated with the development of asthma [56]. Then, our current study further explored the underlying mechanism of curcumin in suppressing airway inflammation in asthma, accumulating more evidence of the role of PPAR*γ* in the pathogenesis of asthma. Meanwhile, it also provides evidence for the further use of curcumin in the treatment of asthma.

## 5. Conclusion

Taken together, our results suggested that OVA- and IL-4-induced airway inflammation and airway mucus hypersecretion were notably blocked by curcumin very likely through a PPAR*γ*-dependent NF-*κ*B signaling pathway in both lung and BEAS-2B cells, indicating that curcumin may be considered an effective therapy for the potential treatment of asthma in the future.

## Data Availability

The data used to support the findings of this study are available from the corresponding author upon request.
